# Preprocessing strategies for plant disease instance segmentation: annotation efficiency, context signals, and dual-metric evaluation on PlantSeg

**DOI:** 10.3389/frai.2026.1835997

**Published:** 2026-10-07

**Authors:** Youssef Natij, Hajar El Karch, Abdelmounaim Belaaribi, Oumaima Lanaya, Meriem El Atik, Abdelkader Mezouari

**Affiliations:** 1 Scientific Research and Innovation Laboratory, Higher School of Technology, Ibn Tofail University, Kénitra, Morocco; 2 Laboratory of Advanced Systems Engineering, National School of Applied Sciences, Ibn Tofail University, Kénitra, Morocco

**Keywords:** annotation efficiency, instance segmentation, plant segmentation, preprocessing strategies, YOLO

## Abstract

Preprocessing choices can strongly affect how well segmentation models localize disease lesions in field imagery, but no systematic instance segmentation study has yet been reported on PlantSeg, the largest publicly available in-the-wild plant disease segmentation dataset (11,458 images; 115 disease categories; 34 host plant species). We evaluated six preprocessing strategies, ranging from annotation-free baselines to expert polygon-guided approaches, each trained for approximately 11.5 h on a single NVIDIA A100 GPU, and assessed them with pixel-level mean intersection over union (mIoU) and instance-level bounding-box and mask average precision (AP). Annotation-guided cropping gave the strongest results: bounding box-guided preprocessing reached 34.12% mIoU and 13.34% mask mAP50, compared with 27.93% and 10.07% for the unprocessed baseline. Context-aware mask augmentation consistently degraded performance, and pixel-level and instance-level metrics ranked the strategies differently. Because the annotation-guided conditions derive their crop windows from ground-truth lesion locations on every split, these gains represent an oracle upper bound on what disease-aware spatial guidance can contribute, not an end-to-end deployable pipeline. The degradation under context-aware augmentation supports the hypothesis that the color and texture contrast at the healthy-to-necrotic boundary carries diagnostic signal the model actively exploits. The divergent metric rankings arise because semantic overlap and instance matching capture complementary properties, which argues for dual-metric reporting in plant disease segmentation. To our knowledge, this is the first systematic instance segmentation study on PlantSeg, and all code and per-instance predictions are publicly released.

## 1 Introduction

Automated plant disease detection is a cornerstone challenge in precision agriculture, with significant implications for global food security (Tilman et al., 2011; FAO, 2021). Annual crop losses attributed to plant diseases exceed $220 billion worldwide (Savary et al., 2019; Strange and Scott, 2005). While classification-based approaches have matured considerably over the past decade (Mohanty et al., 2016; Ferentinos, 2018; Kamilaris and Prenafeta-Boldú, 2018), the field increasingly demands spatially precise outputs: not merely *what* disease is present, but *where* on the plant tissue it manifests and *how much* area it occupies. Instance segmentation, which requires simultaneous detection, localization, and pixel-level delineation of every individual lesion, directly addresses this need.

The transition from classification to instance segmentation introduces new challenges beyond those of recognition alone. Standard segmentation benchmarks have relied on semantic metrics (most commonly mean Intersection over Union, mIoU), which aggregate pixel-level correctness across classes. A missed lesion does contribute false-negative pixels and therefore does lower class IoU; the limitation of mIoU is more specific than a failure to notice misses—it scores pixel agreement only, and is blind to *instance identity*: it does not evaluate how many distinct lesions exist, nor does it penalize split errors (one lesion predicted as several) or merge errors (several lesions predicted as one) (Garcia-Garcia et al., 2017). Instance-level metrics such as AP require every individual lesion to be separately detected, localized, and classified above an IoU threshold, and are sensitive to exactly those instance-structural errors. The two families are therefore complementary rather than redundant, and a model's standing can differ appreciably between them—a distinction that has not been examined in the plant disease segmentation literature (Rottmann et al., 2021).

A further bottleneck concerns preprocessing and annotation cost. Expert polygon annotation of lesion boundaries is expensive and time-consuming. Practically motivated alternatives, such as using bounding box annotations or annotation-free spatial cropping heuristics, could dramatically lower deployment barriers—but their comparative value has not been rigorously quantified on a challenging in-the-wild benchmark.

PlantSeg (Wei et al., 2026) addresses the realism gap by providing 11,458 in-the-wild images across 115 disease categories and 34 host plants, together with expert-annotated polygon masks and derived bounding box annotations. However, all prior work on PlantSeg has relied on semantic segmentation and reported only mIoU, leaving instance-level AP evaluation unexplored.

This study addresses both gaps. We design and evaluate six preprocessing strategies of increasing annotation cost on YOLOv11m-seg (Jocher and Chaurasia, 2024), a modern instance segmentation architecture, and evaluate all strategies on both mIoU and instance-level AP. Our central contributions are:

1. We present, to our knowledge, the first **systematic instance segmentation study** on PlantSeg, reporting bounding-box AP and instance-mask AP alongside the semantic mIoU used by prior work.
2. We quantify the contribution of **disease-aware spatial guidance** under a controlled, one-factor-at-a-time design: annotation-guided cropping improves mIoU by +6.19 pp and Mask mAP50 by +3.3 pp over the unprocessed baseline. Because the crop is derived from ground-truth lesion positions on all splits, we report this explicitly as an **oracle upper bound** on the achievable gain, and identify a learned region-proposal front-end as the route to realizing it in deployment.
3. We show that **suppressing the healthy-to-necrotic tissue boundary degrades performance** on both mIoU and AP relative to mask-guided preprocessing that preserves it, and we advance—as a hypothesis requiring dedicated ablation—the interpretation that this boundary carries actively exploited diagnostic signal.
4. We provide **evidence that mIoU and instance AP rank preprocessing strategies differently**, motivating dual-metric reporting because semantic overlap and instance matching measure complementary properties.

Figure 1 summarizes the overall framework: the six preprocessing strategies, the shared YOLOv11m-seg backbone and training budget, and the complementary pixel-level and instancelevel evaluation applied to every strategy.

**Figure 1:**
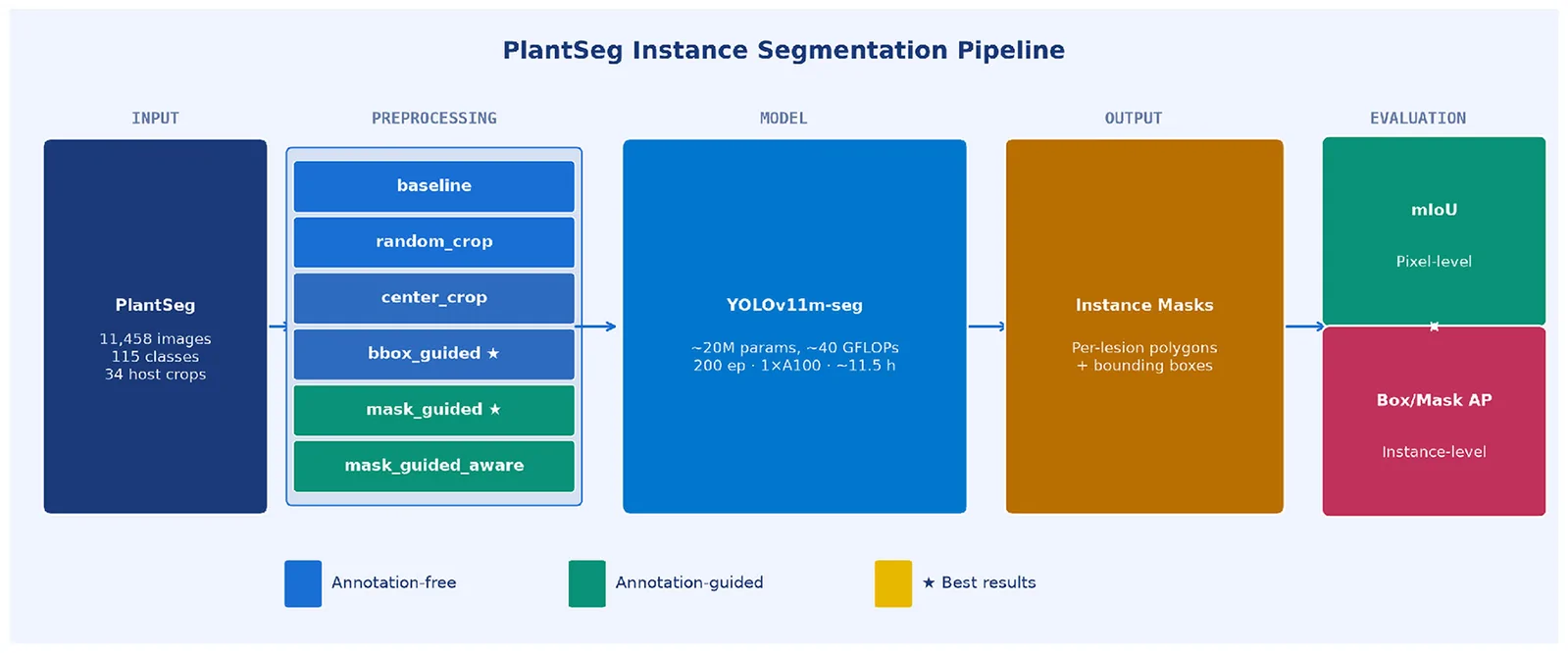
System architecture of the proposed preprocessing evaluation framework. Six strategies of increasing annotation cost are compared using YOLOv11m-seg trained for 200 epochs on a single NVIDIA A100 GPU. All strategies are evaluated using complementary pixel-level (mIoU) and instance-level (Box and Mask AP) metrics.

## 2 Related work

### 2.1 Deep learning for plant disease recognition

Convolutional neural networks have become the dominant paradigm for image-based plant disease diagnosis (Lu et al., 2017; Liu and Wang, 2021). Seminal works by Mohanty et al. (2016) and Ferentinos (2018) demonstrated high accuracy on the PlantVillage laboratory dataset (Hughes and Salathé, 2015), while subsequent studies exposed the gap between controlled and field performance (Barbedo, 2016, 2019; Noyan, 2022). Object detection architectures have been adapted for agricultural tasks, with YOLO-family models emerging as particularly effective due to their balance of speed and accuracy (Pan et al., 2023; Rana and Vaidya, 2025; Miao et al., 2025). The recent YOLOv11 series (Jocher and Chaurasia, 2024) introduced the C3K2 block and C2PSA attention mechanism, improving small-object detection performance relevant to early-stage lesion identification. Lightweight adaptations for field-deployed edge devices have also attracted growing interest (Nyakuri et al., 2025; Bhagat et al., 2024). Recent work has extended this trend to instance segmentation, but large-scale in-the-wild benchmarks remain underexplored. Notably, recent work by Sun et al. (2025) achieves strong binary lesion segmentation on PlantSeg (mIoU 72.84%), though this addresses the two-class formulation rather than the 115-class disease categorization evaluated here.

### 2.2 Instance segmentation architectures

Modern instance segmentation builds on detection-centric architectures. Mask R-CNN (He et al., 2017) introduced a canonical two-stage framework coupling region proposal with mask prediction. Single-stage alternatives such as SOLO (Wang et al., 2020), CondInst (Tian et al., 2020), and YOLACT (Bolya et al., 2019) trade accuracy for speed, with YOLO-family models progressively integrating segmentation heads (Redmon et al., 2016; Jocher et al., 2023; Jocher and Chaurasia, 2024). Transformer-based methods, including the Slice Attention Network (SAN) (Xu et al., 2023), have achieved strong semantic segmentation performance but require substantially higher computational budgets. On the PlantSeg benchmark, (Wei et al. 2026) evaluated DeepLabv3 and DeepLabv3+ variants alongside SAN, SegNeXt (Guo et al., 2022), and ConvNeXt-L (Liu et al., 2022), reporting semantic mIoU up to 53.89% with SegNeXt and 46.24% with ConvNeXt-L. While Wei et al. (2026) reported Mask R-CNN results as part of dataset validation, no prior work has systematically benchmarked instance segmentation with instance-level AP evaluation or investigated the effect of preprocessing strategies on instance-level performance on PlantSeg.

### 2.3 Preprocessing and data augmentation in agricultural vision

Preprocessing and augmentation strategies are critical in low-data regimes common to agricultural AI. Standard spatial augmentations (random flipping, random cropping) have welldocumented effects on object detection models (Zoph et al., 2020; Cubuk et al., 2020), but their interaction with disease-specific spatial signals has not been studied systematically. Disease symptoms are spatially localized—lesions occupy a small, non-uniform portion of the image—suggesting that annotation-guided cropping may offer advantages over random approaches. Wu et al. (2023) explored domain-aware augmentation for lab-to-field transfer, and Xu P. et al. (2023) examined data efficiency under annotation constraints. Several works have used bounding box annotations as a proxy for polygon masks (Fan et al., 2020; Khoreva et al., 2017), motivating the weak supervision setting explored in this paper. Context retention has also been identified as important in medical image segmentation (Chen et al., 2021), suggesting that preserving the boundary between healthy and diseased tissue may be beneficial. Disease severity estimation—quantifying the proportion of plant area affected—similarly benefits from spatially precise segmentation under natural field conditions.

### 2.4 Evaluation metrics for segmentation

The divergence between semantic and instance metrics is well recognized in general computer vision (Garcia-Garcia et al., 2017; Cordts et al., 2016) but has not been examined in plant disease segmentation. mIoU measures pixellevel class agreement averaged over classes; classes absent from both prediction and ground truth are excluded, making the metric forgiving toward missed detections (Rottmann et al., 2021). Instance-level AP, by contrast, requires each individual instance to be predicted with IoU ≥ 0.50 against a ground truth annotation; missed instances directly reduce recall and thus AP. This fundamental difference motivates our dual-metric evaluation protocol.

## 3 Methodology

### 3.1 Dataset

We conduct all experiments on PlantSeg (Wei et al., 2026), the largest in-the-wild plant disease segmentation dataset currently available. It comprises 11,458 images covering 115 disease categories across 34 host plant species, with a mean of approximately 104 images per class. The dataset was collected under natural field conditions, featuring complex backgrounds, variable lighting, partial occlusions, and significant morphological variation between and within classes. Both expert-annotated polygon masks and derived bounding box annotations are provided. We use the dataset's official partition without modification: 9,166 training, 1,146 validation, and 1,146 test images, i.e., an 80*/*10*/*10 split (Table 1). All 11,458 images are accounted for (9,166 + 1,146 + 1,146 = 11,458), and the 1,146-image test partition is the evaluation set underlying every result reported in this paper.

**Table 1:** PlantSeg dataset summary.

Split	Images	Share	Classes	Hosts	Avg. images/class
Train	9,166	80%	115	34	~80
Validation	1,146	10%	115	34	~10
Test	1,146	10%	115	34	~10
Total	11,458	100%	115	34	~100

Splits are the dataset's official 80/10/10 partition and sum exactly to the 11,458 released images. Class and host counts denote the categories represented in each split.

In contrast to commonly used benchmarks, PlantSeg is explicitly designed to resist background bias. PlantVillage (Hughes and Salathé, 2015) (38 classes, lab conditions) and PlantDoc (Singh et al., 2020) (27 classes) are captured in controlled settings, making them substantially less representative of practical deployment conditions. PlantSeg's in-the-wild nature, combined with its scale and annotation richness, makes it the appropriate choice for studying preprocessing strategies under realistic complexity. Figure 2 reproduces the dataset's three-stage construction pipeline (image acquisition, expert-reviewed cleaning, and reviewed polygon annotation), which underpins the annotation quality our guided strategies depend on.

**Figure 2:**
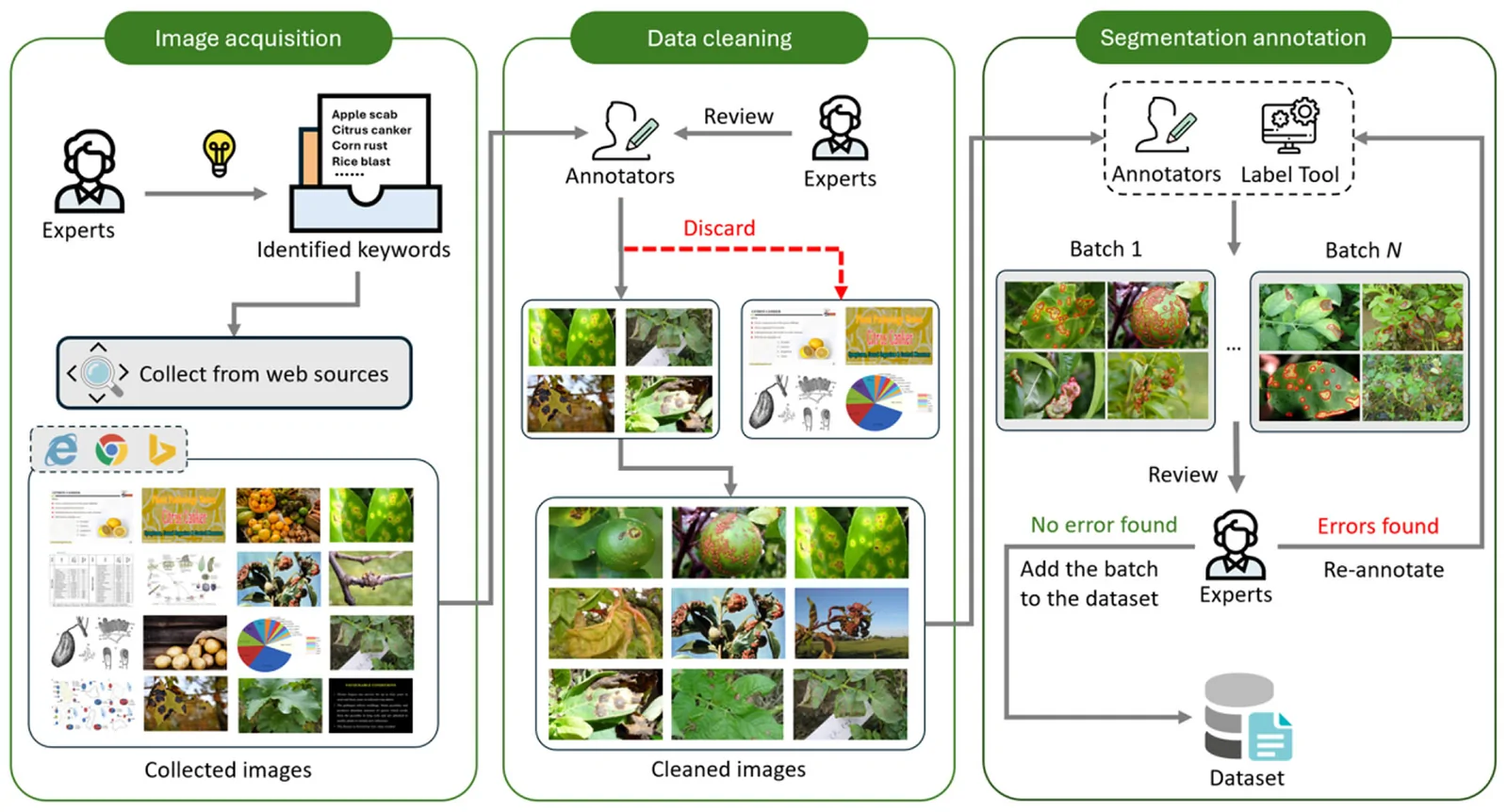
PlantSeg dataset construction pipeline (Wei et al., 2026). The process consists of three stages: **(1) Image acquisition**—domain experts identify disease keywords used to collect images from web sources; **(2) Data cleaning**—annotators and experts jointly review and discard non-conforming images; **(3) Segmentation annotation**—annotators use a labeling tool to produce polygon masks in batches, which are reviewed by experts and re-annotated if errors are found before being added to the final dataset.

### 3.2 Model architecture and training configuration

We use YOLOv11m-seg (Jocher and Chaurasia, 2024), a medium-scale instance segmentation model from the YOLO family with approximately 20M parameters and 40 GFLOPs. The model combines a detection head for bounding box prediction with a segmentation head that generates instance masks via prototype decomposition, enabling single-stage instance segmentation. Note that the segmentation head is trained against ground-truth polygon masks, and this is true of every strategy evaluated here (Section 3.3). All models were pretrained on COCO (Lin et al., 2014) and fine-tuned on PlantSeg for 200 epochs on a single NVIDIA A100 40GB GPU, requiring approximately 11.5 h per strategy (confirmed from training logs; ~41,400–42,400 s). We do not alter the architecture between strategies; all differences stem exclusively from preprocessing.

Shared training hyperparameters for all strategies: input resolution 640 × 640; batch size 64; SGD optimiser with momentum 0.937 and weight decay 5 × 10^−4^; patience 25 (early stopping). The learning rate follows a cosine schedule with initial value η_0_ = 1 × 10^−2^ and final-value factor η_*f*_= 0.01, so that the learning rate decays from 1 × 10^−2^ to η_0_η_*f*_= 1 × 10^−4^ over the 200 epochs (preceded by a standard 3-epoch linear warm-up; the logged effective rate rises to = 3.3 × 10^−3^ at the end of warm-up and decays to = 1.5 × 10^−4^ at epoch 200). Built-in augmentations shared across all strategies: horizontal flip, HSV jitter (*H* = 0.015, *S* = 0.7, *V* = 0.4), translation 0.1, scale jitter 0.5, mosaic probability 1.0, and mixup 0.1.

### 3.3 Preprocessing strategies

We designed six preprocessing strategies to systematically test the effect of annotation type, spatial guidance, and context on segmentation performance. The strategies form a spectrum from annotation-free spatial heuristics to expert polygon-guided approaches.

#### 3.3.1 Baseline

Standard YOLO preprocessing with no spatial modification: images are letterboxed to the target resolution with default augmentations. This serves as the control condition.

#### 3.3.2 Random crop

A naive augmentation approach in which a random sub-region [a random scale in (0.5, 1.0) of the shorter image side at a random location] is cropped before resizing. This increases data diversity but makes no use of disease location information, frequently cropping away the lesion of interest.

#### 3.3.3 Center crop

A fixed central crop [the largest centered square, i.e., a min(*H,W*) × min(*H,W*) region] is extracted from each image before resizing. This reduces background area but assumes that disease features are centrally located, which is not generally true for in-the-wild imagery.

##### 3.3.3.1 Empty-crop exclusion (annotation-free strategies only)

The two annotation-free cropping strategies can, by construction, produce a crop window in which *every* annotated instance falls entirely outside the retained region. Such an image carries no valid segmentation target after cropping: there is no foreground instance to predict and no ground-truth mask against which a prediction could be scored. Following standard practice, these degenerate samples are excluded from the corresponding evaluation split, which reduces the effective test partition to *N* = 1,119 for random_crop and *N* = 1,135 for center–_crop (vs. *N* = 1,146 for all strategies that retain at least one instance per image). We note that the excluded images are, on average, those in which lesions are small or peripheral—i.e., intrinsically harder cases. Their removal can therefore only *inflate* the apparent performance of random_crop and center_crop; the conclusion that annotation-guided strategies are superior is consequently conservative, since the annotation-guided strategies are scored on the full, harder test set while the two annotation-free croppers are scored on an easier subset. Annotation-guided strategies (bbox_guided, mask_guided, mask_guided_aware) never trigger this exclusion because the crop window is defined *from* the annotation and is therefore guaranteed to contain it.

#### 3.3.4 Bounding box-guided (bbox-_guided)

We use the provided bounding box annotations to compute a disease-aware crop region. The crop is centred on the bounding box centroid and extended by a fixed context margin to retain surrounding tissue. Only bounding box labels are consulted *to define the crop window*—see the scope note below, which delimits precisely what this does and does not imply about annotation cost.

##### 3.3.4.1 What bbox_guided does and does not economize

A clarification is required to avoid overstating the annotation-cost result. YOLOv11m-seg has a mask head, and its segmentation loss is computed against ground-truth instance masks. Polygon annotations are therefore used to *train* every strategy in this study, bbox_guided included; we do not employ weakly supervised mask generation, and we make no claim that instance segmentation can be trained from boxes alone. The controlled variable that bbox_guided isolates is narrower and purely a preprocessing question: whether the *crop window* can be derived from cheap axis-aligned boxes instead of expert polygons. Our finding is that it can, at negligible cost in accuracy. The practical implication is correspondingly narrow—if polygons already exist for training, deriving crops from boxes loses nothing—and it does not license the stronger reading that polygon annotation is unnecessary for instance segmentation.

#### 3.3.5 Mask-guided (mask_guided)

We use the expert polygon masks to compute a tight crop around the annotated lesion boundary, with the same context margin as bbox_guided. This retains the full perceptual transition from healthy to necrotic tissue. This is the highest annotation-cost strategy, requiring full polygon annotation.

#### 3.3.6 Context-aware mask augmentation (mask_guided_aware)

An extension of mask_guided that additionally applies a context-suppression augmentation: the healthy tissue surrounding the lesion boundary is blurred or desaturated to force the model to focus on the lesion interior. This strategy tests whether the healthy-to-necrotic boundary is beneficial or irrelevant for learning.

Algorithm 1 provides a complete, reproducible specification of the annotation-guided unioncropping procedure, organized into five phases (annotation → boxes, union region, bypass test, padded crop, optional context suppression). A single *mode* switch selects the annotation source and the optional context-suppression step, so the identical pipeline realizes bbox_guided, mask_guided, and mask_guided_aware and the three differ only in the controlled factor under study.

Algorithm 1 **Annotation-guided union cropping preprocessing (shared by bbox_guided, mask_guided, mask_guided_aware).**

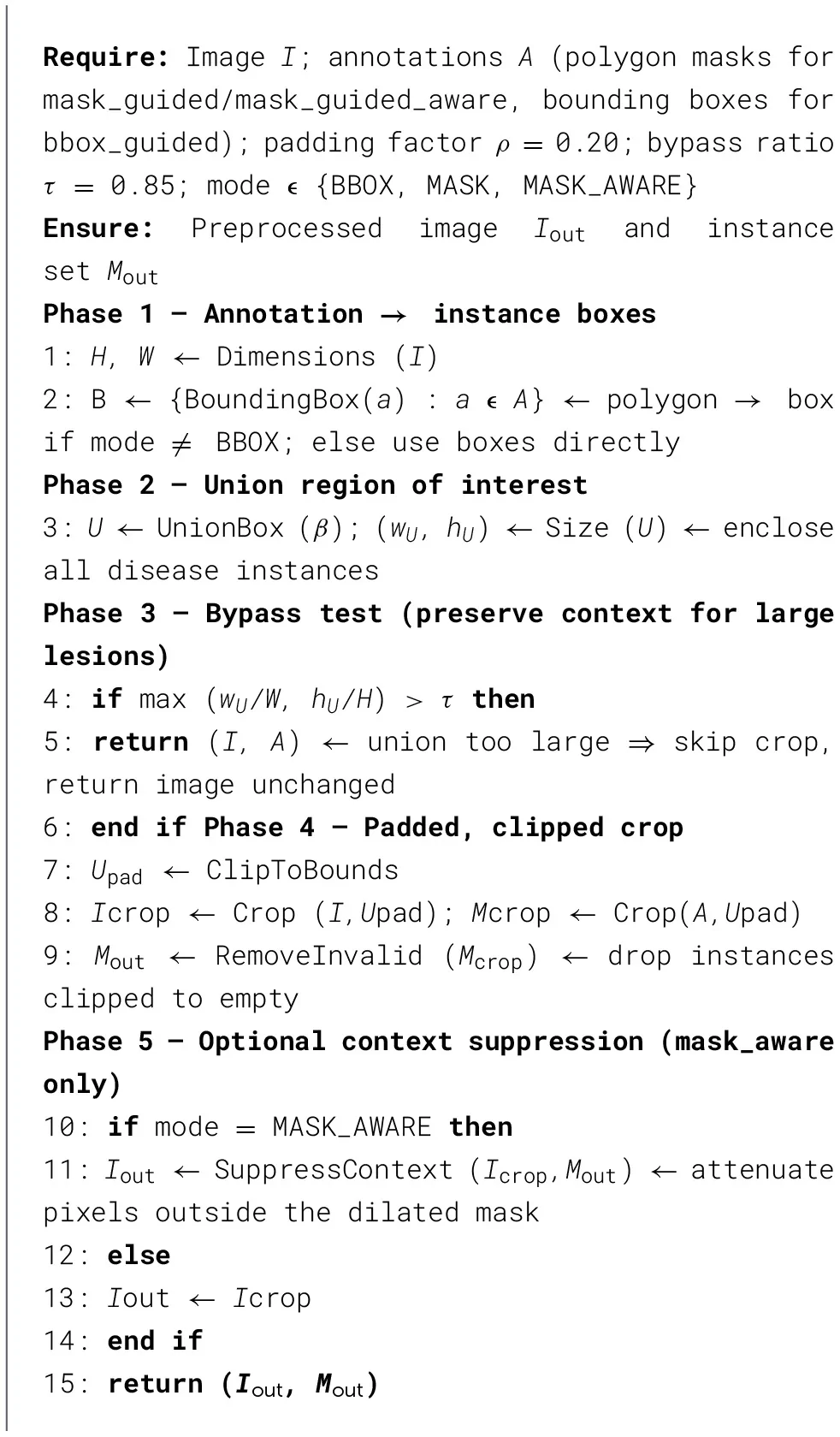

### 3.4 Preprocessing implementation details

For full reproducibility we specify the exact preprocessing behavior. The crop margin (context padding) is a fixed fraction ρ = 0.20 of the union-box width and height, applied symmetrically and then clipped to the image bounds (Algorithm 1, lines 14–15). The bypass threshold is τ = 0.85: if the (un-padded) union box already spans more than 85% of either image dimension, the image is passed through uncropped to avoid degenerate near-full-frame crops. bbox_guided uses the identical procedure but constructs the union box from the provided axisaligned bounding-box annotations rather than from polygon-derived boxes; all other parameters (ρ, τ) are unchanged, so the two guided strategies differ *only* in annotation source.

#### 3.4.1 Split-specific behavior

The annotation-guided crop is computed deterministically from the annotations and is therefore applied identically to the train, validation, and test splits. The annotation-free croppers behave as follows: random_crop samples a fresh random window every epoch on the training split (data augmentation) but uses a single fixed seeded window on validation/test for deterministic, reproducible evaluation; center_crop and baseline are deterministic on all splits. Built-in photometric/geometric augmentations (flip, HSV, mosaic, mixup, scale) are applied to the training split only, identically across all six strategies.

##### 3.4.1.1 Oracle scope of the annotation-guided conditions

Applying the annotation-guided crop to the evaluation splits has a consequence that must be stated plainly, because it bounds how every annotation-guided number in this paper may be read. The crop window is a function of the ground-truth annotation of the very image being evaluated. This is not cross-sample leakage in the conventional sense—the crop geometry depends only on that image's own annotation, never on other images' labels and never on the model—but it is nonetheless *privileged information*: at test time the pipeline is told, by the ground truth, approximately where the lesions are. A deployed system processing a genuinely unseen image does not have this information, and could not compute the crop.

We therefore label the bbox_guided, mask_guided, and mask_guided_aware results throughout this paper as an **oracle upper bound**: they quantify how much performance is available *if* lesion locations were known, and thus upper-bound what a real two-stage system (a learned region proposer followed by this segmentation stage) could achieve. They are not end-to-end results on unseen full images and should not be read as deployment figures. Section 5 states the corresponding limitation, and the train-only-cropping experiment that would convert this bound into a deployable result—annotation-guided cropping applied to the training split alone, with unmodified full images at validation and test—is identified there as the primary next step.

Two of the paper's findings are, by construction, unaffected by this scope condition, and we flag them here because they carry over unchanged. Finding 2 (context suppression, Section 4.5) contrasts mask_guided_aware against mask_guided: both receive *identical* annotation-guided crops, so the oracle information is a shared constant that cancels in the comparison, and the measured difference is attributable to the augmentation alone. Finding 3 (metric divergence) is a statement about the relative behavior of evaluation metrics rather than about absolute attainable accuracy, and the strategy exhibiting it most clearly (random_crop) is annotationfree.

#### 3.4.2 Context-suppression augmentation (mask_guided_aware)

On top of the mask-guided crop, the region outside the dilated polygon mask is attenuated to suppress the healthy-tissue context. We disclose that the precise attenuation parameters (Gaussian blur kernel and desaturation factor) are defined in the released preprocessing code rather than reproduced numerically here, because the manuscript artifact does not bundle the dataset-side pipeline; the reference implementation is provided in the public code release (Section 6.1) to guarantee exact reproducibility. The qualitative effect—removal of the healthy-to-necrotic boundary signal—is the controlled variable studied in Finding 2.

### 3.5 Evaluation protocol

We evaluate all strategies with three families of metric. Because this is an instance segmentation study, we distinguish *mask* AP from *box* AP explicitly and consistently, and we treat mask AP as the primary indicator of instance segmentation quality; box AP is reported as a supporting measure of detection performance. We avoid the unqualified term “mAP50” throughout: every AP value reported in the text, captions, and figures is labeled either **Box mAP50** or **Mask mAP50**. The sole exception is Table 2, whose column headers are disambiguated by the spanning **Box AP** / **Mask AP** group headings above them.

**Table 2:** Test set performance of six preprocessing strategies on PlantSeg (YOLOv11m-seg, 200 epochs, single A100 40GB).

Strategy	Annotation	mIoU ↑	Box AP		Mask AP		P	R	*N*
			mAP50	mAP_50 − 95_	mAP50	mAP_50 − 95_			
mask_guided^†^	Polygonmask	33.72%	**32.69%**	19.95%	**13.70%**	5.47%	35.0%	36.7%	1,146
**bbox_guided** ^†^	Bounding box	**34.12%**	32.25%	**20.12%**	13.34%	**5.61%**	32.8%	**40.1%**	1,146
mask_guided_aware^†^	Polygon + aug.	31.65%	31.28%	19.22%	12.73%	5.14%	**38.0%**	36.2%	1,146
center_crop	None	31.59%	30.48%	19.08%	12.74%	5.30%	35.1%	36.6%	1,135
baseline	None	27.93%	29.83%	17.57%	10.07%	3.97%	30.2%	36.5%	1,146
random_crop	None	30.22%	28.96%	17.62%	12.33%	4.87%	31.5%	37.1%	1,119

We report instance-mask segmentation AP (Mask mAP50, Mask mAP_50 − 95_) as the primary instance segmentation measure and bounding-box detection AP (Box mAP50, Box mAP_50 − 95_) as a supporting measure; mIoU is computed via class-union projection from the instance predictions and is included for comparability with prior PlantSeg work. P/R are box precision/recall. Rows marked^†^ are **oracle** conditions: their crop window is derived from the ground-truth annotation of the evaluated image, so these figures upperbound the achievable gain rather than describing an end-to-end deployable pipeline (Section 3.4). N varies for random_crop and center_crop because images in which all instances fall outside the crop are degenerate and excluded (Section 3.3); comparisons involving those two rows are therefore made on a slightly easier subset. Best value per column in bold.

#### 3.5.1 Mask AP (primary)

Instance segmentation metric computed on predicted instance *masks*. A prediction is matched to a ground-truth instance when their mask IoU meets the threshold; per-class AP is averaged over the 115 classes. We report Mask mAP50 (threshold 0.50) and the stricter Mask mAP_50 − 95_ (averaged over thresholds 0.50–0.95 in 0.05 increments). These quantify how well individual lesions are delineated as distinct objects, and are the metrics against which an instance segmentation method should chiefly be judged.

#### 3.5.2 Box AP (supporting)

The same matching procedure applied to predicted bounding boxes rather than masks, reported as Box mAP50 and Box mAP_50 − 95_. Box AP isolates detection and localization quality from mask-delineation quality; a large Box-to-Mask AP gap indicates that lesions are being found but imprecisely outlined.

#### 3.5.3 mIoU (semantic, for comparability with prior work)

Computed by projecting instance prediction masks to semantic label maps via class-union projection, then averaging perclass IoU over classes present in the test set; classes absent from both prediction and ground truth are excluded. We report mIoU because all prior PlantSeg work reports it, and comparability matters. Its scope should be understood precisely: mIoU measures pixel agreement per class, so a missed lesion *does* register as false-negative pixels and *does* depress class IoU. What mIoU does not represent is instance structure—it is invariant to how the correct pixels are partitioned into objects, and therefore does not register instance count, split errors, or merge errors, all of which AP penalizes.

The two families are thus complementary rather than competing: they answer different questions (how much diseased area is correctly labeled, vs. how well individual lesions are separated and delineated), and reporting both exposes cases where a strategy is strong on one and weak on the other.

## 4 Results

### 4.1 Primary strategy comparison

Table 2 and Figures 3, 4 present the full comparison of all six strategies on the PlantSeg test set.

**Figure 3:**
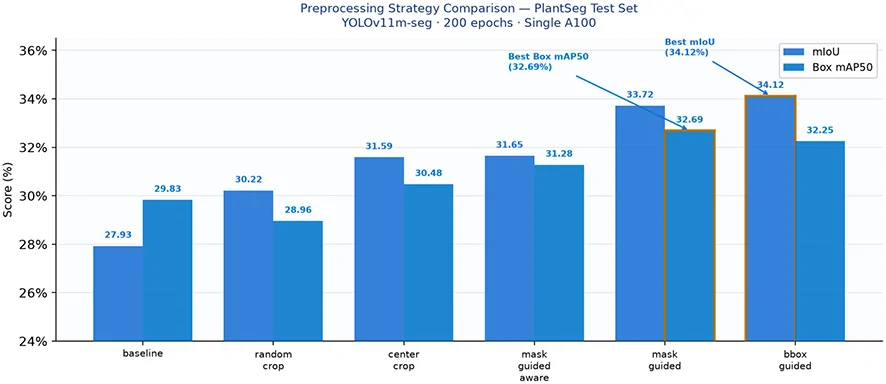
Side-by-side comparison of all six preprocessing strategies on mIoU (blue) and Box mAP50 (cyan). Highlighted borders mark the best result per metric: bbox_guided achieves the highest mIoU (34.12%) and mask_guided the highest Box mAP50 (32.69%); the two are separated by under 0.5pp and are treated as a near-tie (Finding 1).

**Figure 4:**
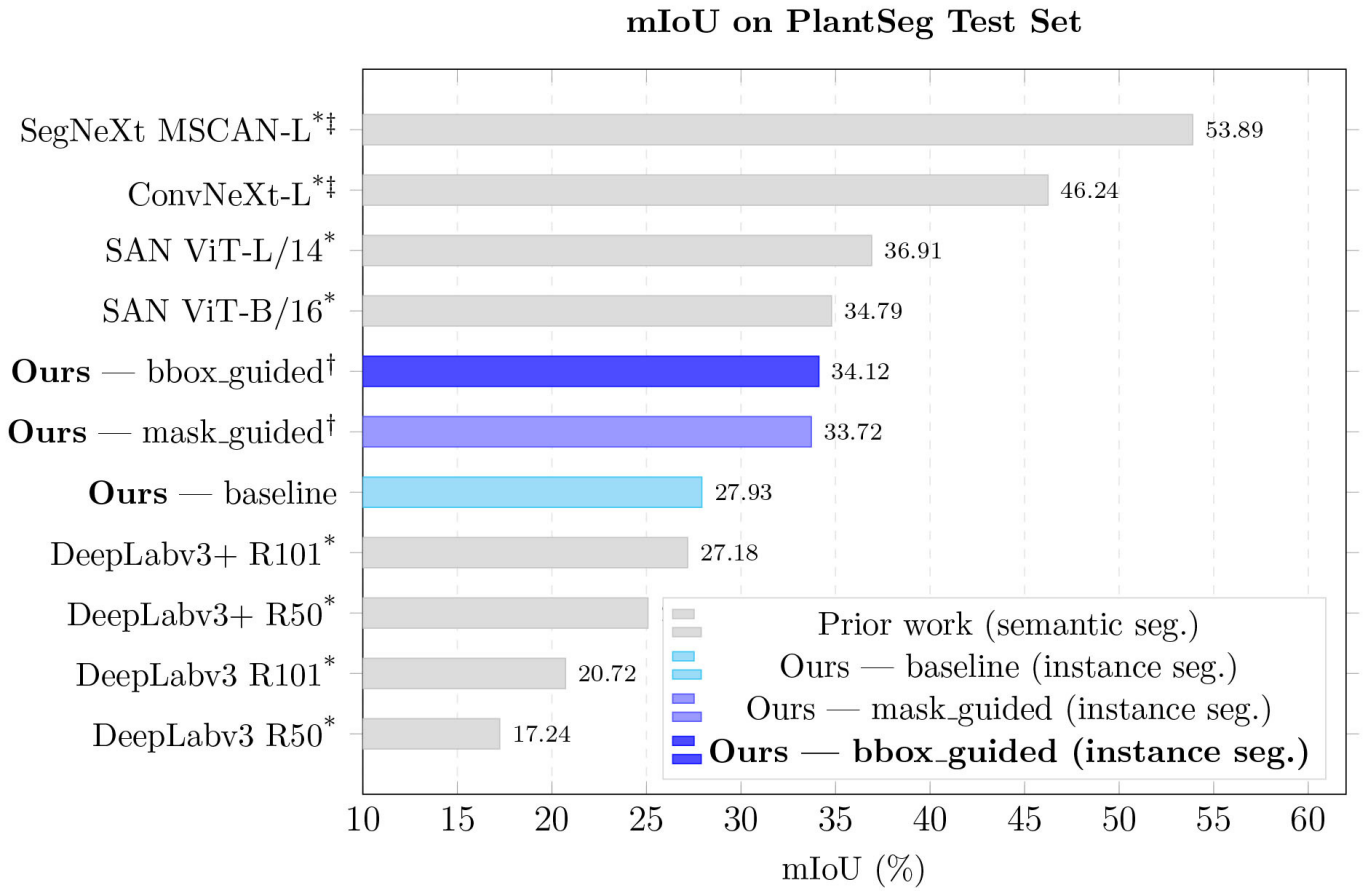
Published PlantSeg mIoU values shown together for context. Our best oracle-cropped strategy (bbox_guided, dark blue) records 34.12%. *This is not a like-for-like ranking*: the gray bars are semantic segmentation models evaluated under their own protocols on unmodified images, whereas our annotation-guided bars are oracle conditions cropped using ground-truth lesion locations (Section 3.4). Relative bar heights should therefore not be read as evidence that one method outperforms another; see Table 4.^†^Oracle condition: crop derived from the ground-truth annotation of the evaluated image (as in Tables 2, 4). *Evaluated on PlantSeg by Wei et al. (2026).^‡^Uses multi-GPU training.

The annotation-guided strategies (bbox_guided and mask_guided) outperform annotationfree approaches on every metric. Between the two, bbox_guided is nominally ahead on mIoU (34.12%) and mask_guided on Box mAP50 (32.69%), but every margin separating them is below 0.5 pp; we treat this as a statistical near-tie rather than a split decision, for the reasons set out in Finding 1 (Section 4.5). The substantial and consistent effect is against the annotation-free strategies: both guided strategies exceed the baseline by +6.19 percentage points (pp) on mIoU and the next-best annotation-free strategy (center_crop) by +2.53 pp on mIoU. Throughout, “pp” denotes an absolute difference in a percentage-valued metric, distinct from a relative percentage change.

#### 4.1.1 Instance-mask quality

Mask AP is the primary instance segmentation measure in this study, since neither semantic mIoU nor box AP directly assesses the quality of the predicted instance *masks* (Table 2). The mask metrics place the two annotation-guided strategies at the top of the ranking: mask_guided is highest on Mask mAP50 (13.70%) and bbox_guided on the stricter Mask mAP_50 − 95_ (5.61%), against 10.07% / 3.97% for the baseline—a +3.6 pp / +1.6 pp improvement mirroring the box-AP and mIoU gains.

The absolute mask-AP values warrant comment, because they are low in absolute terms and considerably below the corresponding box AP (13.70% Mask mAP50 vs. 32.69% Box mAP50 for mask_guided). This gap is informative rather than incidental: lesions are being detected and coarsely localized far more reliably than they are precisely delineated. In-the-wild lesion boundaries are diffuse, irregular, and frequently ambiguous even to expert annotators, and 115-way fine-grained disease classification compounds the difficulty. Readers should therefore calibrate expectations to the mask numbers, which represent the genuine state of instance-level performance on this benchmark, rather than to the more flattering box figures.

#### 4.1.2 Consistency across metrics—and one instructive exception

**Three** of our four evaluation criteria (mIoU, Box AP, Mask AP) rank the strategies consistently, placing annotationguided preprocessing above annotation-free preprocessing. We draw explicit attention to the fact that the fourth, mAcc, does *not*: on the test split the baseline attains the highest mAcc of any strategy (34.33%) while ranking last on mIoU. We analyse this inversion in Section 4.2, and we flag it here so that the corroboration claim is stated accurately—the ordering is stable across three metrics, not uniformly across all four. This tempers, without overturning, the interpretation of the narrow bbox_guided-vs.-mask_guided margin: the categorical advantage of annotation-guided preprocessing over the baseline (+6.19 pp mIoU, +3.3 pp Mask mAP50) is corroborated by pixel-, box-, and mask-level measures, whereas the sub-half-point separation *between* the two guided strategies is not resolved by any of them and is treated as a near-tie throughout.

### 4.2 Mean class accuracy (mAcc)

Table 3 reports mean class accuracy (mAcc) on both the validation and test splits. mAcc is computed as the per-class average of pixel-level accuracy and is more sensitive to class-level correctness than mIoU, since it does not discount for false positives via the union term. This last property is what makes mAcc behave differently from the other three metrics here, and we analyse the resulting inversion below rather than setting it aside.

**Table 3:** Mean class accuracy (mAcc) on the PlantSeg validation and test sets.

Strategy	Val mAcc ↑	Test mAcc ↑	Diff. from Baseline (Val)
mask_guided	**37.67%**	31.77%	+3.32 pp
mask_guided_aware	**37.67%**	31.77%	+3.32 pp
bbox_guided	35.77%	30.67%	+1.42 pp
baseline	34.35%	**34.33%**	—
random_crop	32.37%	30.75%	−1.98 pp
center_crop	30.97%	32.44%	−3.38 pp

“Diff. from Baseline (Val)” is the signed difference in validation mAcc relative to the baseline strategy. Note two features discussed in the text: the baseline attains the highest test mAcc despite ranking last on mIoU, and mask_guided and mask_guided_aware return numerically identical mAcc on both splits—a coincidence we flag as unverified rather than interpret (Section 4.2). Bold values indicate the maximum values.

Three patterns in Table 3 deserve comment, and we treat the second and third as caveats against our own thesis rather than as incidental detail.

First, on the *validation* split the annotation-guided strategies (mask_guided, mask_guided_aware, bbox_guided) rank first through third, consistent with the mIoU ordering.

Second, and contrary to that ordering, **the baseline achieves the highest** *test* **mAcc of any strategy** (34.33%), while ranking last on mIoU (27.93%). mAcc is thus the one metric of the four that does not corroborate the paper's central comparison, and we state that plainly rather than reporting only the three that agree. The mechanism is visible in the val-totest columns: the annotation-guided strategies degrade sharply between splits (bbox_guided −5.1 pp, 35.77 → 30.67; mask_guided −5.9 pp, 37.67 → 31.77) whereas the baseline is essentially flat (−0.02 pp). Because mAcc omits the union term, it rewards liberal, high-recall pixel labeling and does not discount the resulting false positives; the baseline, which sees whole uncropped images and predicts diffusely, is favored under precisely that scoring rule, while the guided strategies—optimized on tightly cropped, lesion-centred views—transfer less evenly to it. The honest reading is that annotation-guided preprocessing improves localization and delineation quality (mIoU, Box AP, Mask AP) but confers no advantage in per-class pixel recall, and may cost some. This is a genuine boundary on the scope of our claim.

Third, mask_guided and mask_guided_aware return *numerically identical* mAcc on both splits (37.67% and 31.77%), despite differing on every other metric. Two strategies agreeing to four significant figures on two independent splits is more consistent with an artifact of the mAcc computation—for instance a cached or shared intermediate in that evaluation path—than with a substantive equivalence. We previously read this as evidence that context suppression leaves class-level recognition untouched; we withdraw that interpretation, since the data do not support distinguishing it from a computational artifact. Pending a re-run of the mAcc evaluation, these two cells should be treated as unverified, and no conclusion in this paper rests on them.

The broader methodological point stands independently of the resolution: reporting several metrics is valuable precisely because they can disagree, and a study reporting only mIoU—or only mAcc—would have presented a misleadingly uniform picture of this comparison.

### 4.3 Qualitative analysis

Figure 5 presents representative inference examples comparing the baseline (no preprocessing) against our best-performing mask-guided strategy across two disease classes at opposite ends of the severity spectrum.

**Figure 5:**
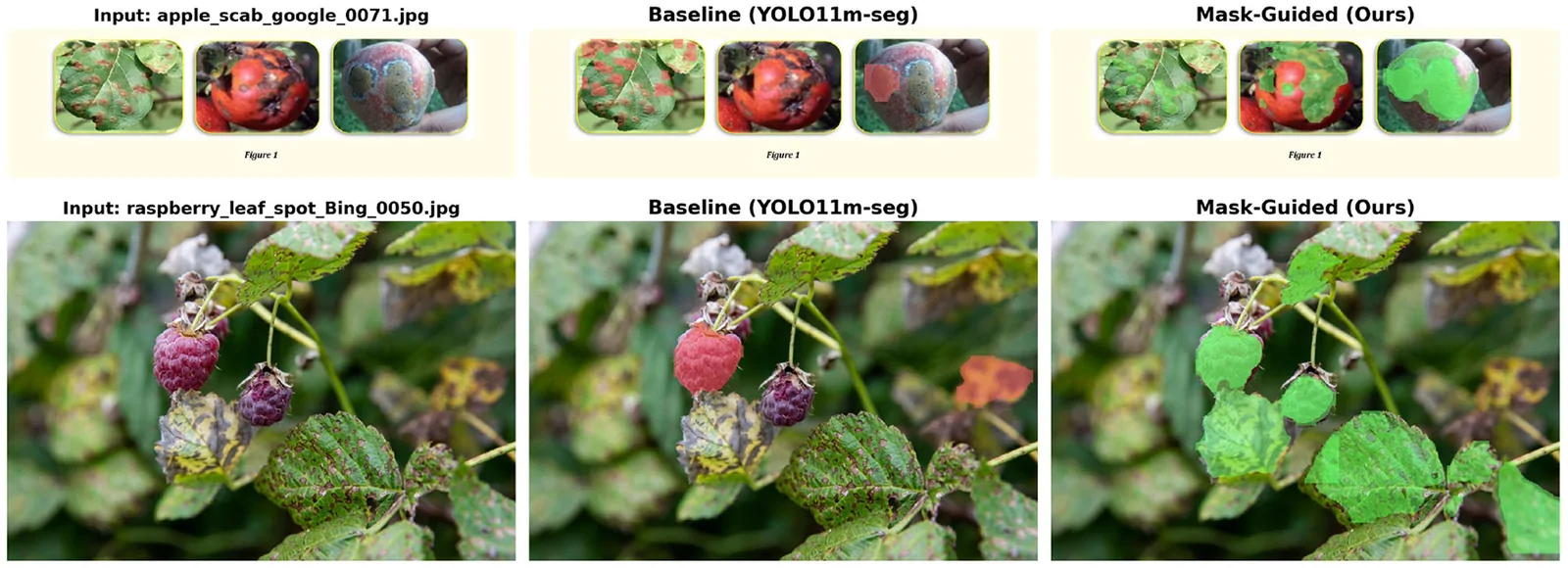
Qualitative comparison of Baseline vs. Mask-Guided (ours) on two PlantSeg test images. **Top**: apple scab. The baseline produces a partial, boundary-misaligned mask on the primary lesion; the mask-guided model correctly segments both instances including the characteristic dark-border necrotic zone. **Bottom**: raspberry leaf spot. The baseline fails to detect the subtler leaf lesions entirely; the mask-guided model successfully localizes and segments the affected foliage. Each panel shows: input image **(left)**, baseline prediction (center), mask-guided prediction **(right)**. Green overlays represent predicted instance segmentation masks.

These examples are consistent with the quantitative findings: the mask-guided crop focuses the model on the lesion-containing region during training, sharpening its sensitivity to earlystage and low-contrast disease symptoms. The baseline, trained on full-resolution letterboxed images dominated by background context, lacks the spatial inductive bias needed to localize subtle lesions.

We caution against over-reading these panels. They are two hand-picked cases in which the guided model performs well, they do not display ground-truth masks alongside the predictions, and two examples cannot represent behavior across 115 classes and 34 hosts. Without the ground-truth overlay a reader cannot assess false positives, false negatives, or boundary error from these figures, and the low absolute Mask AP of Table 2 guarantees that failures are common even though none is shown here. A qualitative evaluation adequate to the claim would present original image, ground truth, and each strategy's prediction in a shared coordinate frame, and would deliberately include failure modes—small and peripheral lesions, occlusion, adjacent instances, visually similar diseases, and cluttered backgrounds—rather than favorable cases only. We regard the present figure as illustrative of the mechanism rather than as evidence of typical performance, and note the extended comparison as outstanding work in Section 5.

### 4.4 Comparison with prior semantic segmentation baselines

Table 4 lists published mIoU figures for prior PlantSeg models (Wei et al., 2026) alongside our own. We present these as a **contextual reference only**, and explicitly not as a like-for-like comparison, for four reasons that we ask readers to keep in view.

**Table 4:** Published PlantSeg mIoU figures (Wei et al., 2026) shown alongside ours as a **contextual reference, not a like-for-like comparison**.

Method	Backbone	Params	GFLOPs	mIoU	Box mAP50	Mask mAP50
Prior work—semantic segmentation, different protocol (Wei et al., 2026)						
DeepLabv3 (Chen et al., 2017)	ResNet-50	40M	65G	17.24%	—	—
DeepLabv3 (Chen et al., 2017)	ResNet-101	59M	85G	20.72%	—	—
DeepLabv3+ (Chen et al., 2018)	ResNet-50	43M	67G	25.08%	—	—
DeepLabv3+ (Chen et al., 2018)	ResNet-101	62M	88G	27.18%	—	—
SAN (Xu et al., 2023)	ViT-B/16	86M	195G	34.79%	—	—
SAN (Xu et al., 2023)	ViT-L/14	307M	810G	36.91%	—	—
ConvNeXt-L (Liu et al., 2022)	ConvNeXt-L	~234M	~210G	46.24%	—	—
SegNeXt (Guo et al., 2022)	MSCAN-L	49M	70G	53.89%	—	—
This work — instance segmentation, single A100, ~11.5h / strategy						
Ours — baseline	YOLOv11m-seg	~20M	~40G	27.93%	29.83%	10.07%
Ours — bbox_guided^†^	YOLOv11m-seg	~20M	~40G	34.12%	32.25%	13.34%
Ours — mask_guided^†^	YOLOv11m-seg	~20M	~40G	33.72%	32.69%	13.70%

The prior models perform semantic segmentation under their own training and evaluation protocols (resolution, augmentation, schedule, and evaluation code were not held constant with ours, and we did not re-run them); our rows perform instance segmentation. Rows marked ^†^ are oracle conditions whose crop derives from ground-truth lesion locations, whereas prior models were evaluated on unmodified images. Parameter and GFLOP values for prior work are estimates from the original architecture papers, not measured under a common harness; training hardware and time were not reported by the PlantSeg authors and are omitted. **No claim of surpassing or matching these models is made or implied**; establishing that requires same-protocol instance segmentation baselines (Section 5). Instance metrics are unavailable (–) for semantic models by construction.

First, the task differs: the prior models perform semantic segmentation and do not separate individual instances, whereas ours performs instance segmentation; mIoU is the only metric on which the two can be placed side by side at all, and it is the metric least suited to describing what our model does. Second, the training and evaluation protocols differ—resolution, augmentation, optimiser schedule, and evaluation implementation were not held constant, and we did not re-run those models under our protocol. Third, and most importantly, our annotationguided rows are oracle conditions (Section 3.4): their crops derive from ground-truth lesion locations, while the prior models were evaluated on unmodified images. Fourth, parameter and GFLOP figures for prior work are estimates taken from the original architecture papers rather than measured under a common harness.

We therefore make *no claim* to surpass, match, or close a gap to any of these models, and readers should not infer a ranking from the table. Establishing where our approach genuinely stands requires same-protocol instance segmentation baselines—Mask R-CNN and other YOLO segmentation variants trained and evaluated on this identical split under a full-image test protocol—which we identify in Section 5 as a necessary next step. Table 4 serves only to situate the absolute magnitude of PlantSeg mIoU values for a reader unfamiliar with the benchmark's difficulty.

Figure 6 plots the same mIoU values against parameter count. Read with the caveats above, it and Table 4 support one narrow, defensible observation and no more: competitivemagnitude PlantSeg mIoU values are reachable at ~20M parameters on a single GPU, whereas the reference points from prior work sit at 40–307M and, for the strongest two, required multiGPU training. We regard parameter and infrastructure economy as the meaningful contribution here, since both are measured directly and are protocol-independent. Accuracy standing is a separate question that this table cannot settle: our annotation-guided figures are oraclecropped, the prior figures come from a different task and protocol, and the gap to SegNeXt (53.89%) and ConvNeXt-L (46.24%) is in any case substantial. Whether a ~20M-parameter instance segmentation model is genuinely competitive on PlantSeg must be settled against same-protocol instance segmentation baselines, which we have not run.

**Figure 6:**
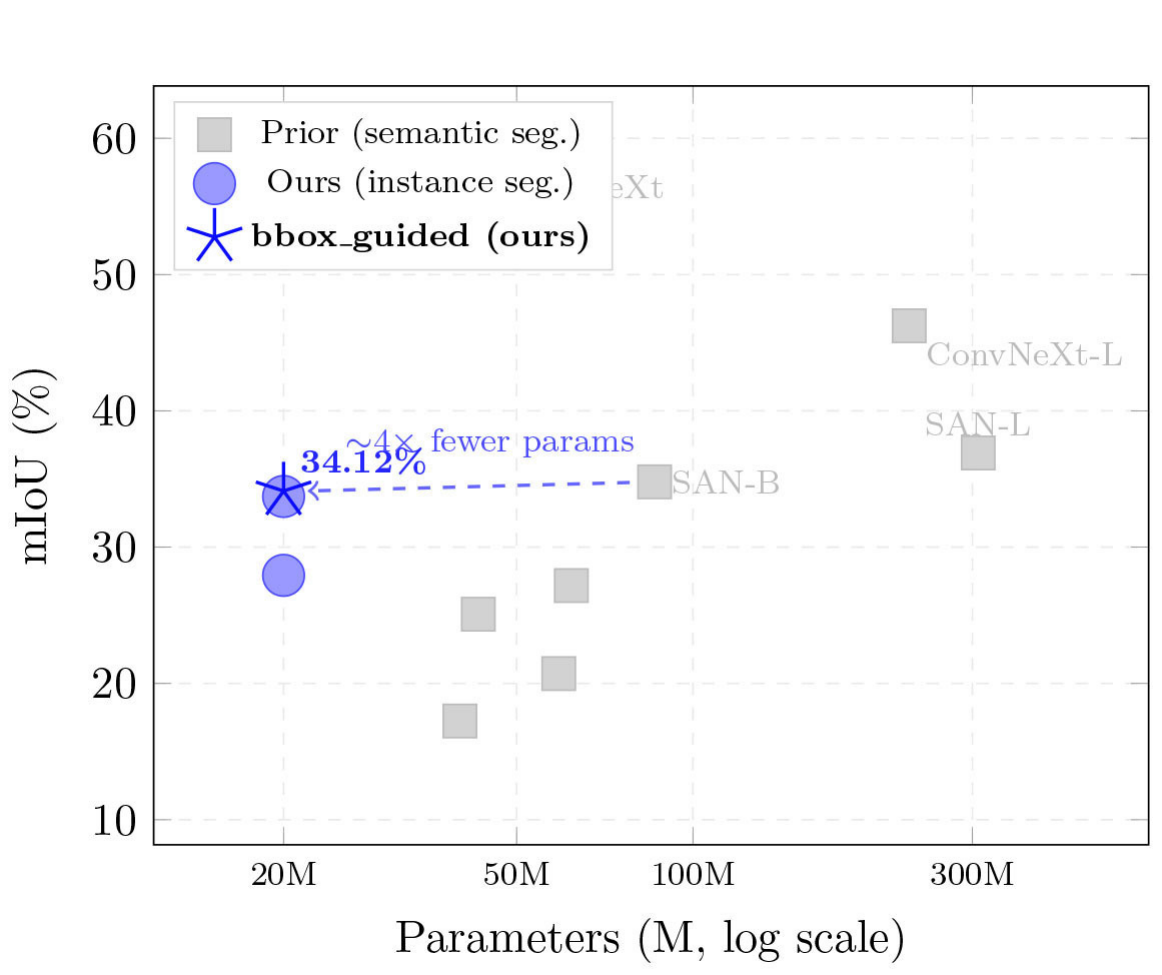
mIoU vs. parameter count (log scale). Our YOLOv11m-seg models occupy the low-parameter region (~20M) of the plot, roughly 4 × below SAN ViT-B/16 (~86M) and an order of magnitude below ConvNeXt-L (~234M) and SAN ViT-L/14 (~307M); all our runs used a single GPU. The dashed arrow marks this parameter-count difference. As in Figure 4, vertical position is *not* a fair performance ranking: prior points are semantic segmentation under different protocols on unmodified images, while our annotation-guided points are oracle conditions. The plot is included to convey the parameter-scale contrast, not to assert competitive accuracy.

### 4.5 The four key findings

#### 4.5.1 Finding 1: a near-tie between the two guided strategies

bbox_guided is nominally ahead on mIoU (34.12% vs. 33.72%) and mask_guided on Box mAP50 (32.69% vs. 32.25%) and Mask mAP50 (13.70% vs. 13.34%). Every one of these margins is below 0.5 pp, and with a single training seed and no variance estimate we cannot establish that any of them is larger than run-to-run noise. We therefore characterize this as a **statistical near-tie**, not a dual-winner result, and we do not recommend one strategy over the other on the basis of these differences. The practically useful statement is the weaker one: deriving the crop from cheap bounding boxes costs nothing measurable relative to deriving it from expert polygons, so on this evidence the choice can be made on annotation-cost grounds alone. Establishing a genuine ordering would require the multi-seed protocol described in Section 5.

#### 4.5.2 Finding 2: the context signal

mask_guided_aware underperforms mask_guided despite receiving identical spatial guidance, differing only by the added context-suppression augmentation (31.65% vs. 33.72% mIoU; 31.28% vs. 32.69% Box mAP50; 12.73% vs. 13.70% Mask mAP50). The degradation is consistent across all three metrics, and the magnitude (−2.07 pp mIoU) is several times the near-tie margins discussed above. This is also the one comparison in the paper immune to the oracle caveat, since both arms use the same ground-truth-derived crop, which cancels.

Our interpretation—that the color and texture contrast at the healthy-to-necrotic boundary is an actively exploited diagnostic feature rather than background noise—is a *hypothesis consistent with* this result, not a demonstrated mechanism. The augmentation changes several things simultaneously: it blurs, it desaturates, it introduces artificial edges at the dilation boundary, and it shifts the image statistics away from those of the validation and test data. Any of these could account for the drop without the boundary carrying special diagnostic content. Distinguishing among them requires the factorized ablation (blur alone, desaturation alone, varied boundary widths, varied augmentation strength) set out in Section 5. What the experiment establishes securely is narrower and still useful for practitioners: *suppressing lesionsurrounding context, as implemented here, measurably hurts*, so augmentations of this family should be validated rather than assumed beneficial.

#### 4.5.3 Finding 3: the random_crop divergence

random_crop ranks last of six on Box mAP50 (28.96%) yet fifth on mIoU (30.22%), placing it *above* the baseline on the pixel metric while it is the worst strategy for instance detection. This is our clearest evidence that mIoU and instance AP capture complementary properties. The mechanism is consistent with the metric definitions given in Section 4: resampling a fresh window each epoch exposes the model to diverse partial views, which aggregates into reasonable per-class pixel agreement, while routinely truncating or excluding lesions degrades the instance structure that AP measures—objects are split across crop boundaries, merged, or missed entirely. A study reporting mIoU alone would rank random_crop mid-field and miss that it is the weakest option for any instance-level application. We note this comparison carries the caveat of Section 3.3: random_crop is scored on a slightly easier *N* = 1,119 subset, which if anything flatters it and strengthens the point.

#### 4.5.4 Finding 4: cheap boxes suffice to define the crop

bbox_guided matches mask_guided to within 0.5 pp on every metric (Finding 1), despite deriving its crop window from axis-aligned boxes rather than expert polygons. Bounding box annotation requires substantially less expert time than polygon annotation (Khoreva et al., 2017), so where crop derivation is concerned the cheaper annotation is sufficient.

We are deliberately precise about the scope of this claim, which is narrower than an annotation-efficiency result for instance segmentation as such. As set out in Section 3.3, YOLOv11m-seg trains its mask head against ground-truth polygon masks in *all* strategies, bbox_guided included. Polygons are therefore not eliminated from the pipeline; they are eliminated only from the *preprocessing* step. The result says that if polygons already exist for training, nothing is lost by using boxes to compute crops—and, more speculatively, that a weakly supervised variant dispensing with polygons entirely is worth investigating. It does not say that instance segmentation can be trained without polygon supervision, which we did not test.

### 4.6 Metric divergence analysis

Table 5 and Figure 7 summarize the ranking of each strategy under both metrics, illustrating the systematic divergence between mIoU and Box mAP50 rankings.

**Table 5:** Ranking divergence across preprocessing strategies under mIoU and Box mAP50.

Strategy	mIoU Rank	Box mAP50 Rank	Δ Rank	mIoU / Box mAP50
bbox_guided	1st	2nd	+1	34.12% / 32.25%
mask_guided	2nd	1st	−1	33.72% / 32.69%
mask_guided aware	3rd	3rd	0	31.65% / 31.28%
center_crop	4th	4th	0	31.59% / 30.48%
random_crop	5th	6th	+1	30.22% / 28.96%
baseline	6th	5th	−1	27.93% / 29.83%

The random_crop divergence (5th on mIoU, last on Box mAP50) is the clearest instance. Rank changes among the closely spaced middle entries should be read with the near-tie caveat of Finding 1, as several separations are well under 1 pp. random_crop ranks 5th on mIoU but last on Box mAP50 — clearest evidence of metric divergence.

**Figure 7:**
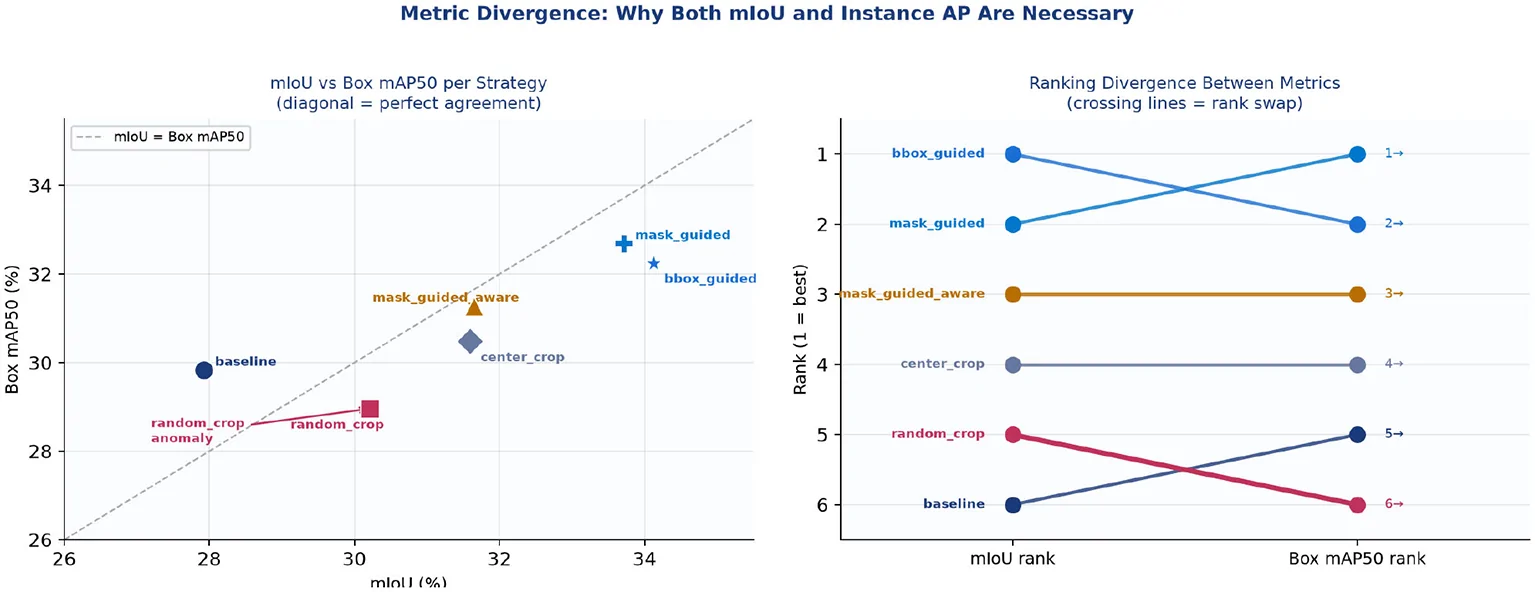
Metric divergence analysis. **Left**: mIoU vs. Box mAP50 per strategy; points below the diagonal score relatively better on Box mAP50. **Right**: bump chart of rank position under each metric, where crossing lines indicate rank swaps. The bold rose line marks the random_crop divergence (5th on mIoU, last on Box mAP50), the clearest evidence that semantic overlap and instance matching capture complementary properties.

Four of the six strategies change rank position between the two metrics. The most striking divergence is random_crop, which appears adequate under mIoU (ranking above baseline) while being the worst-performing strategy on instance detection. This has a direct methodological implication: prior PlantSeg benchmarks, which report mIoU exclusively, may have unknowingly favored strategies that perform well on pixel coverage while failing on instance-level tasks.

### 4.7 Per-class prediction behavior

PlantSeg's 115 classes and 34 hosts make global averages a partial picture. As a classlevel analysis we examined the per-class prediction distribution of every strategy (Figure 8). Two observations hold across all strategies. First, *class coverage is high and strategy-invariant*: every strategy produces confident predictions (≥0.25 score) for 110–112 of the 115 disease classes (95.7–97.4%). The model does not collapse onto a few easy classes under any preprocessing regime; the low absolute mAP therefore reflects *localization and confusion* errors distributed across most classes, not a failure to attempt rare classes. Second, the per-class prediction mass is strongly long-tailed (Gini = 0.38–0.40 for all strategies), closely tracking PlantSeg's intrinsic class imbalance (~104 images/class on average but a heavy tail of rare categories); preprocessing shifts overall accuracy without materially reshaping this class-frequency profile.

**Figure 8:**
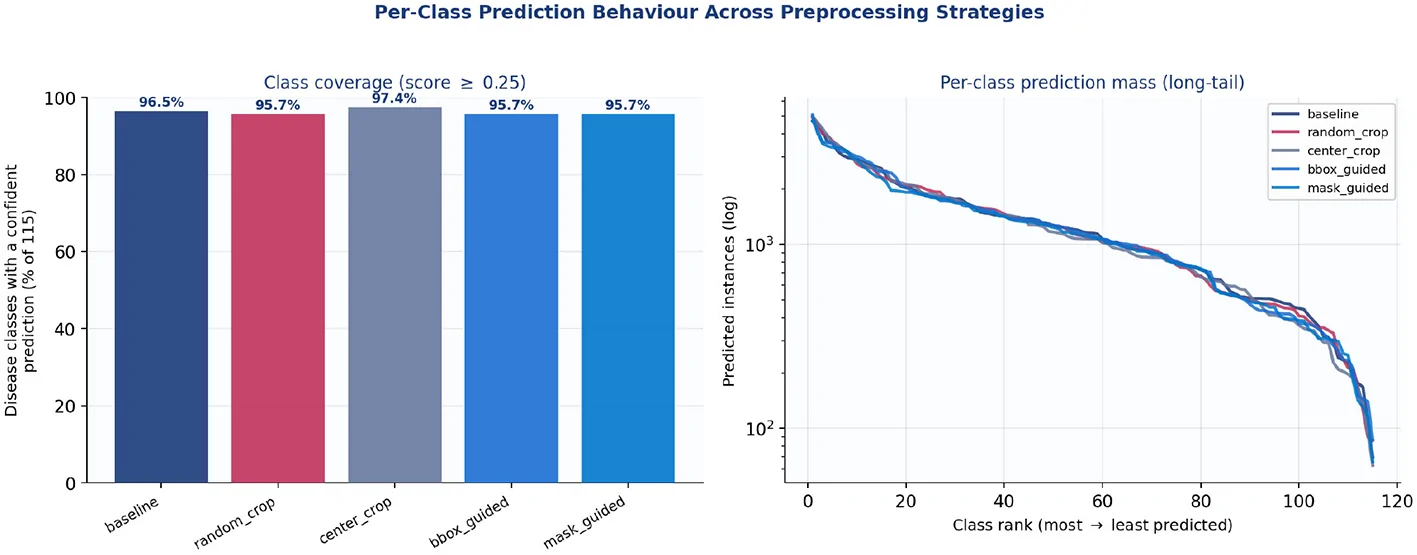
Per-class prediction behavior. **Left**: fraction of the 115 disease classes receiving at least one confident prediction—high (≥95.7%) and essentially invariant to preprocessing. **Right**: per-class predicted-instance count, sorted (log scale), showing a long-tailed profile common to all strategies that mirrors the dataset's class imbalance.

We are explicit about what this analysis does and does not establish. Class coverage counts the classes for which the model emits at least one confident prediction; it therefore rules out one specific failure mode—that a low global average conceals a collapse onto a handful of easy classes—and shows that mode is not occurring. It does *not* demonstrate that those predictions are correct, and it is consequently a much weaker statement than per-class accuracy. A class predicted often and always wrongly is indistinguishable, in this analysis, from a class predicted often and correctly.

A full per-class and per-host stratification of Box AP, Mask AP, recall, and IoU—together with per-class image and instance counts, breakdowns by lesion size, occlusion level, and instance count, and a confusion matrix over commonly conflated disease categories—requires joining predictions to ground-truth labels, which reside with the dataset rather than in the released prediction artifact. That analysis is not included here, and its absence is a real limitation for a 115-class benchmark: our global averages could mask substantial heterogeneity across classes and hosts, and we cannot presently say which disease categories or which host species benefit most from annotation-guided preprocessing. We identify this stratified evaluation, and the confusion analysis in particular, as a priority in Section 5.

### 4.8 Ablation perspective

Although framed as a comparison of six strategies, the experimental design is itself a set of controlled, one-factor-at-a-time ablations: each adjacent pair of strategies isolates a single preprocessing variable while holding the architecture, hyperparameters, seed, and training budget fixed. Reading Table 2 along these axes:

- **Spatial guidance** (baseline → bbox_guided/mask_guided): adding annotation-guided cropping is the single largest factor, contributing +6.19 pp mIoU and +3.6 pp Mask mAP50. This isolates the effect of *where* the model looks.
- **Annotation source** (mask_guided → bbox_guided): replacing polygon-derived crop boxes with cheap bounding-box-derived crop boxes, with ρ and τ held constant, changes mIoU by only +0.40 pp and Mask mAP50 by −0.36 pp. This isolates the value of annotation *precision* and shows it is marginal for preprocessing.
- **Context suppression** (mask_guided → mask_guided_aware): adding the boundarysuppression augmentation while keeping the crop identical costs −2.07 pp mIoU and −0.97 pp Mask mAP50. This isolates the diagnostic value of the healthy-to-necrotic boundary (Finding 2).
- **Guided vs. unguided cropping** (random_crop/center_crop → guided): at matched cropping “effort,” annotation-guided windows outperform annotation-free windows on every metric, isolating the contribution of *disease-awareness* as opposed to cropping *per se*.

These four contrasts already constitute the principal ablations relevant to the paper's claims. We acknowledge that finer parameter sweeps—the padding factor ρ, the bypass ratio τ, the context-margin width, and the augmentation strength in mask_guided_aware—would further enrich the analysis. Each such sweep requires a full multi-day training run per setting on the target hardware; we therefore scope a systematic ρ/τ/margin sensitivity study as the immediate next step (Section 5.5) rather than expand the present controlled comparison, whose factor isolation we consider the more defensible contribution.

### 4.9 Computational cost of the preprocessing choice

We measured inference latency for *all six* preprocessing configurations to determine whether the choice of preprocessing carries a runtime cost. All measurements were taken on the NVIDIA A100 40GB GPU used for training, at 640 × 640 input resolution, averaged over the test split, and decomposed into pre-processing, model inference, and post-processing (NMS).

As Table 6 shows, end-to-end latency is essentially invariant across strategies (10.22–10.44 ms/img, =96–98FPS). Model inference dominates (=91% of the pipeline), with preprocessing and NMS together under 9%. This invariance is the result we draw: because all six strategies share one backbone and one input resolution, preprocessing changes *which* pixels the model sees without changing what inference costs, so adopting annotation-guided preprocessing carries no runtime penalty relative to the baseline.

**Table 6:** Pipeline latency per preprocessing strategy, measured on an NVIDIA A100 40GB GPU at 640 × 640, averaged over the test split.

Strategy	Pre (ms)	Inference (ms)	NMS (ms)	Total (ms)	FPS
baseline	0.13	9.50	0.82	10.44	95.8
random_crop	0.12	9.50	0.67	10.30	97.1
center_crop	0.12	9.45	0.65	10.22	97.8
**bbox_guided**	0.12	9.44	0.72	10.28	97.3
mask_guided	0.12	9.58	0.73	10.43	95.8
mask_guided_aware	0.12	9.45	0.76	10.33	96.8

End-to-end latency is essentially constant across strategies because all share the same backbone and input resolution; the preprocessing choice therefore carries no inference-time cost. These are data-centre GPU figures and do not characterize edge-device performance.

We deliberately refrain from extending this into a claim about edge deployment. These measurements come from a data-centre accelerator, and A100 throughput does not predict performance on the mobile phones, UAV companion computers, or embedded controllers where an agricultural system would actually run: those platforms differ in memory bandwidth, thermal envelope, available precision, and power budget, and the relevant figure there is single-image latency under a power cap rather than batched throughput. Substantiating suitability for such hardware would require measuring batch-size-one latency, peak memory, serialized model size, and power draw on a representative device such as a Jetson Orin, which we have not done. What the model's ~20M parameters and ~40 GFLOPs support is the narrower observation that it is small enough for such an evaluation to be worth attempting—not that it has passed one.

## 5 Discussion

### 5.1 Disease-aware preprocessing as a modeling decision

Our results demonstrate that spatial preprocessing is not a neutral choice but a substantive modeling decision with measurable performance consequences. The transition from annotationfree cropping (random_crop, center_crop, baseline) to annotation-guided cropping (bbox_guided, mask_guided) produces consistent, large improvements (+6 pp on mIoU). This finding is consistent with the broader literature on region-focused training in object detection (Zoph et al., 2020; Cubuk et al., 2020): providing the model with spatially relevant views of the target accelerates learning and improves final accuracy. In the plant disease domain, where symptoms occupy a small, variable fraction of the image and background is highly complex, this effect is amplified. Recent YOLO-based detection studies on in-the-wild agricultural imagery report similar patterns, where annotation-aware training protocols consistently outperform naive augmentation pipelines (Miao et al., 2025).

### 5.2 The lesion boundary: a hypothesis worth testing

The underperformance of mask_guided_aware relative to mask_guided is a robust observation—it is consistent across mIoU, Box AP, and Mask AP, and, uniquely among our comparisons, unaffected by the oracle caveat because both arms share the same crop. The *explanation* for it is not yet established, and we separate the two here.

The interpretation we find most plausible is that the transition zone between healthy and diseased tissue carries diagnostic signal the model exploits. It has domain backing: plant pathologists use this boundary in manual diagnosis, where the transition often carries pathogenspecific signatures such as the chlorotic halo of bacterial infections or the powdery white margin of *Erysiphe*. It also parallels medical imaging, where peri-lesional context is diagnostically informative (Chen et al., 2021). On this reading, suppressing the boundary discards information the model would otherwise use.

That interpretation is, however, under-determined by our experiment, and we present it as a hypothesis rather than a conclusion. Our context-suppression augmentation alters several factors at once—Gaussian blur, desaturation, an artificial edge introduced at the maskdilation boundary, and a shift in image statistics away from the unaugmented validation and test distributions. A simple distribution-shift account, in which any comparable train-time perturbation would have hurt, explains the result equally well and attributes nothing special to the boundary. Adjudicating between these requires factorizing the augmentation: blur alone, desaturation alone, several dilation widths, and several augmentation strengths, each trained separately. Until that is run, the defensible claim is the operational one—context-suppression augmentation of this form degrades performance and should not be adopted without validation—rather than the mechanistic one.

### 5.3 Instance vs. pixel metrics in plant pathology

The evidence that mIoU and instance AP rank preprocessing strategies differently has direct consequences for the plant pathology AI community. Applications involving disease *severity quantification* (e.g., what fraction of the canopy is diseased) are naturally served by pixellevel metrics. Applications involving disease *incidence monitoring* (e.g., how many individual lesions are present, which plants show early-stage symptoms) require instance-level metrics. Publishing only one metric obscures the model's capability profile (Rottmann et al., 2021). We recommend that future plant disease segmentation benchmarks report both mIoU and instance-level AP (mask AP in particular) as complementary, non-redundant indicators.

### 5.4 Practical implications for annotation investment

The near-equivalence of bbox_guided and mask_guided bears on annotation planning, though in a more limited way than it might first appear. Bounding box annotation requires considerably less expert time than polygon annotation (Khoreva et al., 2017), and our results indicate that the cheaper annotation is entirely adequate for *deriving the crop window*: the two strategies differ by under 0.5 pp on every metric, a margin we cannot distinguish from noise.

The limit on this implication bears repeating, because it is easy to overstate in exactly the direction that would flatter the result. Polygon masks supervise the segmentation head in all six strategies, bbox_guided included, so this study does not show that polygon annotation can be dispensed with for training an instance segmentation model. A project that must produce instance masks still needs mask supervision, or a weakly supervised substitute we did not evaluate. What our result supports is that once such annotations exist, no further precision is required for the preprocessing stage—and, prospectively, that pairing box-derived crops with weakly supervised mask generation (Khoreva et al., 2017; Fan et al., 2020) is a promising direction for genuinely annotationlight pipelines. That combination remains untested here. The narrower finding still aligns with broader work on annotation-efficient learning (Xu P. et al., 2023; Mullins et al., 2024).

### 5.5 Limitations and future work

This study has substantive limitations, several of which bound how its results may be used. We state them directly.

#### 5.5.1 Oracle test-time cropping (the principal limitation)

As detailed in Section 3.4, the annotation-guided strategies compute their crop from the ground-truth annotation of the image being evaluated, on validation and test as well as training. Those results therefore describe an upper bound on what disease-aware spatial guidance can contribute when lesion locations are known, and are not end-to-end results on unseen images; a deployed system has no such annotation and could not compute the crop. The comparison between guided and annotation free strategies is confounded accordingly, and no deployment claim should be drawn from it. Two findings are exempt by construction—Finding 2, whose two arms share an identical crop, and Finding 3, which concerns metric behavior and centres on an annotation-free strategy—and these we regard as unaffected. The decisive remedy is to retrain with annotation guided cropping applied to the training split only, evaluating on unmodified full images; if the resulting model still outperforms the baseline, the benefit is real and deployable, and if it does not, the effect is an artifact of test-time cropping. We were unable to run this within our compute budget and place it first among the extensions below. A learned region-proposal front-end, whose predicted regions replace ground-truth crops at inference, is the corresponding architectural route.

#### 5.5.2 Single-seed training and absent significance testing

All experiments use one fixed seed (42), so we report no variance estimates or confidence intervals, and several margins we discuss fall below 0.5 pp—plausibly within run-to-run variation. This is a genuine weakness, mitigated only partially: the seed is held *identical* across all six strategies, so every model shares initialization and data ordering and the preprocessing factor is isolated ceteris paribus; and training runs 200 epochs, well past the patience of 25, with asymptotic behavior over the final ~50 epochs (Figure 9), so differences are unlikely to reflect incomplete training. We distinguish two claims accordingly. The categorical one—annotation-guided cropping beats annotation-free by roughly +6 pp mIoU, corroborated by mIoU, Box AP, and Mask AP—is an order of magnitude beyond plausible seed noise and we consider it secure within the oracle scope above. The ordering *between* the two guided strategies is not established at all, and we describe it throughout as a near-tie. We note also that mAcc does not corroborate the categorical claim (Section 4.2); the corroboration is across three metrics, not four. A multi-seed protocol with mean ± std and paired significance tests, or a paired bootstrap over test images, remains necessary.

**Figure 9:**
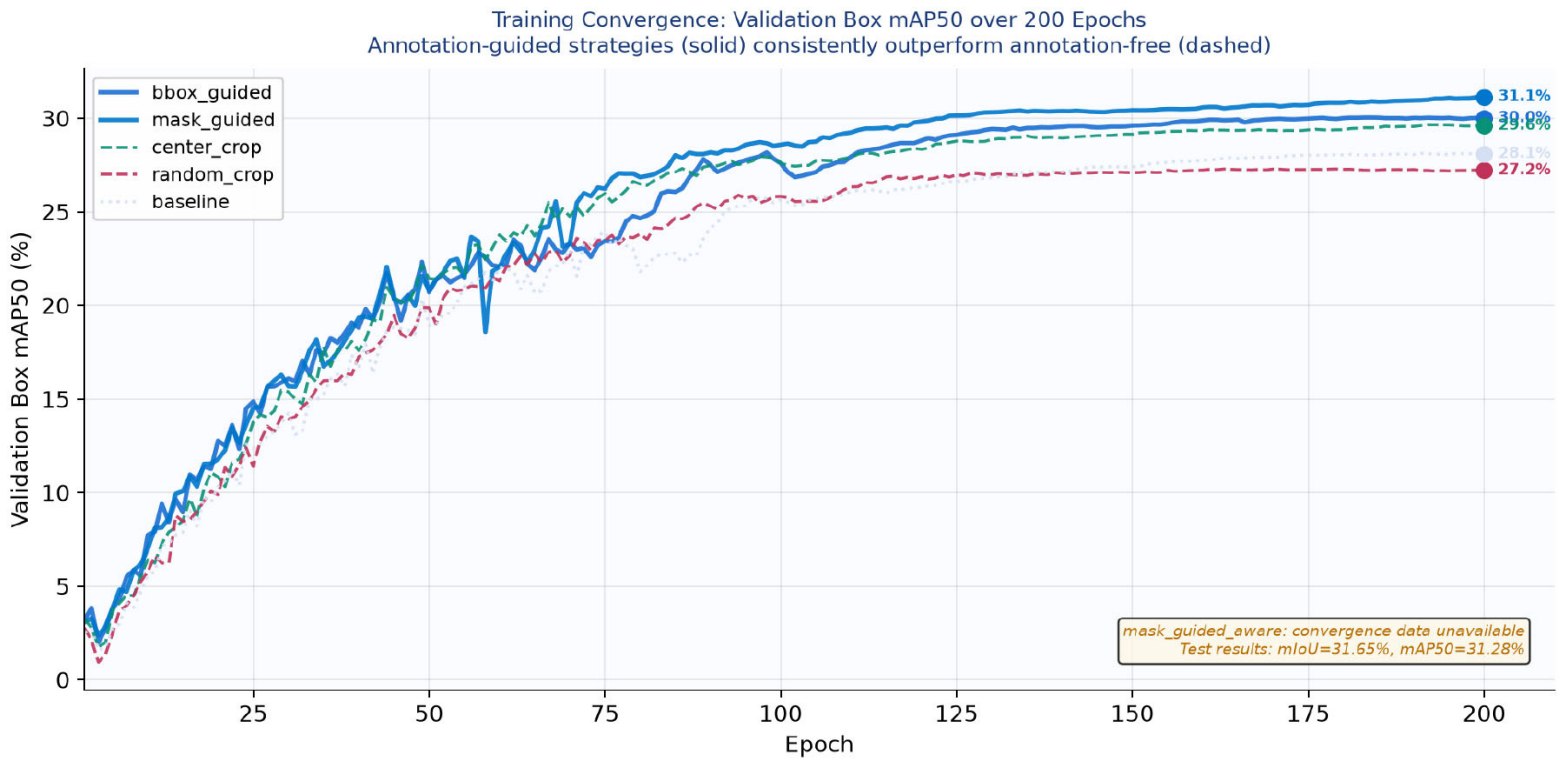
Validation Box mAP50 across 200 training epochs for five preprocessing strategies (from training logs). The annotation-guided strategies (bbox_guided, mask_guided) converge to higher validation performance than the annotation-free ones (baseline, center_crop, random_crop), and all curves are asymptotic over roughly the final 50 epochs, supporting the claim that the reported differences reflect the preprocessing regime rather than incomplete training. *Omission*: the per-epoch log for mask_guided_aware was lost and that curve is absent; its final checkpoint and test metrics were unaffected and are reported in Table 2. Since Finding 2 rests on that strategy, we note explicitly that we can verify its endpoint but not its trajectory, and regenerating the log is part of the artifact audit described in Section 5.

#### 5.5.3 Confounded preprocessing factors

Annotation-guided cropping changes lesion position, lesion scale, background proportion, and the probability that a lesion is visible at all—simultaneously. Our design cannot attribute the observed gain to disease-aware guidance specifically rather than to the smaller effective field of view or higher lesion-to-background ratio that guided cropping also produces. The control that would separate these is a random-crop baseline matched to the guided crop-size distribution, which we did not run. The parameters ρ = 0.20 and τ = 0.85 were likewise fixed a priori and never varied, so we cannot report sensitivity to them.

#### 5.5.4 Unequal effective test sets

random_crop and center_crop are scored on *N* = 1,119 and *N* = 1,135 images respectively, against *N* = 1,146 for the other four, because crops containing no annotated instance are degenerate and excluded (Section 3.3). Strictly, the rankings are therefore not computed on a common test set. The excluded images are those with small or peripheral lesions—the harder cases—so the exclusion favors the two annotation-free croppers and renders our conclusion conservative rather than inflated. A fully rigorous protocol would map predictions back to original image coordinates and count lesions outside the retained crop as missed instances, placing all strategies on one common ground-truth set.

#### 5.5.5 Incomplete class-level and qualitative evaluation

For a 115-class, 34-host benchmark we report only global averages plus a class-coverage statistic that establishes the absence of few-class collapse but not per-class correctness (Section 4.7). Per-class and per-host AP, stratification by lesion size and occlusion, and a confusion matrix over conflated categories are all absent, as is a qualitative comparison showing ground truth and failure cases (Section 4). Global averages may mask considerable heterogeneity.

#### 5.5.6 Artifact gaps

The per-epoch training log for mask_guided_aware was lost (Figure 9), and the identical mAcc values for mask_guided and mask_guided_aware (Section 4.2) are unverified and possibly a computation artifact. The exact context-suppression parameters live in the released preprocessing code rather than in the manuscript. An audit of the evaluation code and logs is warranted before these specific numbers are relied upon.

#### 5.5.7 Scope

Results cover one architecture (YOLOv11m-seg) on one dataset (PlantSeg) with one random seed, and no same-protocol instance segmentation baseline was trained, so we cannot say how our absolute numbers compare with Mask R-CNN or other YOLO segmentation variants under identical conditions.

#### 5.5.8 Future work

In priority order: (1) the train-only-cropping experiment with full-image evaluation, converting the oracle bound into a deployable result, together with a learned regionproposal front-end; (2) same-protocol instance segmentation baselines (Mask R-CNN, YOLOv8seg) on the identical split and full-image protocol; (3) a matched random-crop control plus sensitivity sweeps over ρ, τ, and context-margin width (Section 4.8); (4) multi-seed training with mean ± std and paired significance tests, and re-evaluation on a common test set with out-of-crop instances counted as missed; (5) a factorized ablation of the context-suppression augmentation—blur alone, desaturation alone, varied boundary widths and strengths—to test the boundary-signal hypothesis of Section 5; (6) per-class, per-host, and lesion-size-stratified AP with a confusion matrix, and an expanded qualitative comparison including failure cases; (7) edge-device profiling (batch-one latency, memory, model size, power) on representative hardware such as a Jetson Orin; and (8) extension across model capacities and to domain adaptation settings spanning crop species and geographic regions (Wu et al., 2023).

## 6 Reproducibility details

We record the settings needed to reproduce these experiments. **Model and training**: Ultralytics YOLOv11m-seg initialized from the COCO-pretrained yolo11m-seg.pt checkpoint; 200 epochs; batch size 64; input resolution 640 × 640; SGD with momentum 0.937 and weight decay 5 × 10^−4^; initial learning rate η_0_ = 1 × 10^−2^ with cosine decay and final-value factor η_*f*_= 0.01 (final rate 1 × 10^−4^); early-stopping patience 25 epochs; validation every 5 epochs; random seed 42 for all six runs; single NVIDIA A100 40GB; wall-clock 41,687–42,400s (~11.6 h) per strategy. **Augmentation** (training split only, identical across strategies): horizontal flip; HSV jitter *h* = 0.015, *s* = 0.7, *v* = 0.4; translation 0.1; scale 0.5; rotation 0.0; mosaic 1.0; mixup 0.1. **Preprocessing**: padding factor ρ = 0.20, bypass ratio τ = 0.85 (Algorithm 1). **Data**: PlantSeg official 80*/*10*/*10 split (Table 1); each strategy materializes its own preprocessed copy of all three splits before training.

Several details are not recorded in our released artifacts and we flag them rather than reconstruct them: exact Ultralytics, PyTorch, CUDA, and operating-system versions; the confidence and NMS thresholds used at evaluation (Ultralytics defaults unless overridden); mask output resolution; the checkpoint-selection criterion (best-on-validation vs. last epoch); and the exact per-run completed-epoch count and whether early stopping triggered. The context suppression parameters for mask_guided_aware (Gaussian blur kernel and σ, desaturation factor, mask-dilation width, application probability, and order) are defined in the released preprocessing code but are not reproduced numerically in this manuscript. Readers reproducing Finding 2 in particular should consult that code directly, since the finding depends on those values.

### 6.1 Code and data availability

The full source code for all six preprocessing pipelines, training scripts, and evaluation utilities is publicly available at https://github.com/YouROS12/plantseg-mask-guided, together with per-instance predictions in COCO format and per-epoch training logs for the five strategies whose logs were retained. The PlantSeg dataset is available at https://github.com/tqwei05/PlantSeg (Wei et al., 2026).

## 7 Conclusion

We presented a systematic study of six preprocessing strategies for plant disease instance segmentation on PlantSeg (Wei et al., 2026), the largest in-the-wild plant disease segmentation benchmark available. Using YOLOv11m-seg on a single A100 GPU, and reporting instance-mask AP and box AP alongside the semantic mIoU used by prior work, we obtained four findings, each of which we state together with the conditions under which it holds.

Annotation-guided cropping substantially outperforms annotation-free preprocessing: +6.19 pp mIoU and +3.3 pp Mask mAP50 over the unprocessed baseline, corroborated across mIoU, Box AP, and Mask AP. This gain is measured under an *oracle* condition—the crop derives from ground-truth lesion locations on every split—so it upper-bounds what disease-aware spatial guidance can contribute rather than describing a deployable pipeline. Establishing the deployable portion requires training-only cropping with full-image evaluation, or a learned region proposer, and is the first item of our future work. We make no claim of surpassing prior semantic segmentation models, whose figures we report only as context.

Deriving the crop window from cheap bounding boxes matches deriving it from expert polygons to within 0.5 pp on every metric—a margin we cannot separate from single-seed noise, and which we therefore treat as a near-tie rather than a win for either. Because the segmentation head is trained against polygon masks in all strategies, this economizes the preprocessing stage only, and does not show that instance segmentation can be trained without mask supervision.

Suppressing the healthy-to-necrotic tissue boundary degrades performance consistently across all three metrics. This comparison is unaffected by the oracle caveat, since both arms share an identical crop. That the boundary carries actively exploited diagnostic signal is our hypothesis for why; distribution shift from the augmentation itself remains an equally consistent explanation, and separating them requires the factorized ablation we describe. The supported practical guidance is that context-suppression augmentation should be validated, not assumed beneficial.

The random_crop divergence—fifth on mIoU while last on Box mAP50—shows that semantic overlap and instance matching capture complementary properties: mIoU scores pixel agreement and is blind to instance identity, count, and split or merge errors, which AP penalizes. We recommend that plant disease segmentation work report both, and that instance segmentation studies foreground mask AP, whose absolute values here (10%−14%) describe the task's real difficulty far better than the more flattering box figures.

For practitioners, the actionable guidance is to preserve lesion-surrounding context rather than suppress it, to evaluate on pixel-level and instance-level metrics together, and—where lesion locations can be estimated at inference—to expect meaningful gains from disease-aware spatial guidance, whose realizable magnitude on unseen images remains to be established.

## Data Availability

The original contributions presented in the study are included in the article/supplementary material, further inquiries can be directed to the corresponding author.
